# Ministry of Health Action and Other Matters

**Published:** 1919-10-25

**Authors:** 


### THE FUTURE OF THE BLIND.

### II. Ministry of Health Action and other Matters.

The Advisory Committee on the Welfare of the Blind, being a Standing Committee, made its suggestions or recommendations to its parent, the Local Government Board, from time to time during the course of its labours. What they exactly were is not altogther clear from the annual report of the committee. One dealing with a weekly allowance of money to each of the unemployable blind, to be paid to him in his home, is, it appears, not feasible in the existing state of the law and must be considered after new legislation; others are to be dealt with in the manner described in the Circular on the Welfare of the Blind issued by the Ministry of Health.

Parliament is to be asked to make a grant which, if voted, will be available in respect of the following services: (1) Workshops for the blind; (2) provision of assistance to home-workers; (3) hostels and homes for the blind; (4) home teaching; (5) book production; (6) the work of Counties Associations; and (7) miscellaneous. Regulations governing the distribution of the grant are appended to the circular of the Ministry.

### Blind Persons Working at Home.

A large number of blind persons work in their own homes. This may be for need of neighbouring workshops, because the nature of the work is suited to home industry, for reasons of health, or for other special reasons. These workers—knitters, basket-makers, and the like—need encouragement just as much as do those who work in company with each other, and the Ministry of Health seeks a scheme under which this scattered field of labour may be effectively overlooked and tended. The needs of the home worker are varied. There is the question of tools and equipment, of the supply of materials at the lowest market prices, of assistance in making and finishing articles, of advice as to current prices, of markets for the finished article, of advertising, and of arrangements as to periodic returns as to output.

These conditions equally apply to workshops, where, of course, administration and inspection are much more a matter of the routine work of a perfected system. To the isolated blind home teaching is also recognised as an important matter. The solace of the Braille system of reading and writing can rarely become the possession of a blind person through his own study. The home teacher is necessary, and at present there is a pronounced shortage of teachers. It is work that is peculiarly suited to the educated blind, and in this field the blind may lead the blind with great success. Part of the proposed Government grant will therefore be devoted to helping towards the provision of adequate salaries for blind teachers.

### Book Production.

Another branch noted for a share of the grant is book production. It is proposed to give 2s. 6d. towards the cost of each volume, and 2d. per copy of a magazine, periodical, or sheet of music produced in embossed type by an approved agency. The largest institution which publishes Braille literature is the National Institution for the Blind, who in 1918 issued 17,943 bound volumes, 37,812 magazines, 103,064 newspapers, and nearly 20,000 volumes and pieces of music in Braille type. The same organisation last year produced 12,494 items in Moon type, which is suited to less sensitive fingers or to persons in advanced years learning to read.

Another agency dealing with the embossed word is the National Lending Library for the Blind, through whose agency is distributed on loan a prodigious number of volumes.

### Workshops and Homes.

A grant is proposed at a rate not exceeding £20 per head per annum in respect of the total number of workshop employees in regular employment throughout any period. The recognised standards of the trade in which the workshop employees are engaged so far as they relate to rates of pay, bonus, hours of labour, and holidays must be observed by an agency. In no case may the hours of labour exceed forty-eight hours per week.

In a home, which is defined as a residential institution for the care and maintenance of adult blind persons, or a hostel or residential institution for the provision of board and lodging for blind workers, a grant is proposed of not more than £5 per annum in respect of each blind person regularly resident.

In the circular no mention is made of children, for these come under the provisions of the Education Acts. The proposals of the Ministry of Health deal also with other services mentioned in the first part of this article; clear conditions of the keeping and auditing of accounts, the keeping of registers, and other matters are laid down in the rules and regulations.

### Prevention is Better than Cure.

For many years those interested in the cause of the blind, and in this respect must be especially mentioned Gardener's Trust and its highly respected Secretary, Mr. Henry Wilson, have been struggling to prevent much avoidable blindness, by educating those who needed education in the matter of attention to the eyes of the newly born. The various Midwives Acts have, of course, helped in this direction by improving the status of the midwife. But compulsory notification of ophthalmia neonatorum, which commenced on April 1, 1914, should crown the labours of the past. The report of the Royal Commission on Venereal Diseases gives some statistics in reference to the results obtained through this long-desired and necessary step on the part of the public health authorities.

The war, of course, put back the clock of social reform, and because of shortage of medical men it is probable that notification of cases of ophthalmia neonatorum has not been carried out as completely as will be the case from now on. It is estimated that 70 per cent. of the cases of the disease are due to maternal gonorrhœa, and it has been found that over 30 per cent. of the inmates of the schools for the blind are blinded by neglect and unsuitable treatment of the inflammation of their eyes at the time of birth.

With capable doctors, careful midwives, and instruction as to personal hygiene amongst the masses, there is every hope that the numbers of the blind will decrease, in relation to the extent of the population, year by year. The statistics of child-blindness as brought out by the report of the Advisory Committee on the welfare of the blind are very pathetic. In an analysis of the ages at which blindness occurred the Committee state that the largest group is that under one year, in which the percentage is 21.4. Of these 20.6 became blind within six months of birth, very largely from preventable causes.

The previous article appeared on October 18, p. 55.

